# Causes of Delayed Care Seeking for Chronic Suppurative Otitis Media at a Rwandan Tertiary Hospital

**DOI:** 10.1155/2018/5386217

**Published:** 2018-07-02

**Authors:** Jean Paul Darius Nshimirimana, Kaitesi Batamuliza Mukara

**Affiliations:** ENT Department, College of Medicine and Health Sciences, University of Rwanda, Rwanda

## Abstract

**Background:**

Chronic suppurative otitis media causes serious lifelong consequences when treatment is delayed. Early detection and effective treatment result in a good outcome and possible complications are thus avoided. The aim of this study was to determine the factors resulting in delayed care seeking for treatment of CSOM.

**Method and Patient:**

The study was a cross-sectional survey conducted at a tertiary teaching hospital in Rwanda. A questionnaire was used to collect data of patients diagnosed with CSOM who attended ENT Department during the study period. We defined delayed care seeking as seeking treatment 6 months after onset of symptoms. Data was entered and analysed using SPSS 16.0.

**Result:**

This study enrolled 109 patients, 97 (88.9%) of whom had delays in care seeking. Majority were young adults ranging between 21 and 30 years (39.2%) while 58.8% were rural residents. Fifty-eight patients (56.9%) of those with delayed presentation used traditional medicine. The main reason for delayed care seeking was low knowledge of CSOM reported by 88 (90.7%) patients.

**Conclusion:**

This study shows that majority of patients with delayed care seeking are young adult patients. There is low knowledge concerning this disease and this significantly contributes to delayed care seeking.

## 1. Introduction

Chronic suppurative otitis media (CSOM) is a common condition affecting ears and affects more children than adults [1] although the disease may affect a child and persist into adulthood if left untreated [2]. CSOM usually manifests as intermittent or persistent ear discharge through a perforated tympanic membrane [3–5]. Initially, CSOM presents with mucopurulent discharge through perforated tympanic membrane with otalgia; then hearing impairment follows [6]. Thus, the clinical manifestation of CSOM tends to change as the disease progresses. It is a common reason for patients to consult otorhinolaryngology department in developing countries.

The CSOM is predominantly a disease of the developing counties with the prevalence of 11%, whereas in developed countries it is lesser than 2% [3]. The prevalence of CSOM in Sub-Saharan African countries ranges from 0.4 to 4.2% [2]. A study conducted in Nigeria reported that among patients attending ENT 25% were found to have CSOM [3] while in Tanzanian a survey estimated that 14% of ENT patients had CSOM [2].

Predisposing factors have been frequently attributed to the population with low socioeconomic conditions, overcrowding, poor hygiene, poor nutrition, frequent upper respiratory infection, inadequate antibiotic treatment, and poor and unavailable healthcare [4, 6, 7]. The diagnosis is made from history taking and otoscopic findings. When indicated that a CT-scan should be requested to rule out any associated complications [8, 9].

The goals of management of CSOM are twofold: disease eradication and closures of the tympanic membrane perforation. The treatment of CSOM could be nonsurgical, surgical, or combined modalities [10, 11]. Tympanomastoidectomy should be considered in case of failure of medication or complicated CSOM [2, 10, 12]. Untreated disease is associated with life threatening complications including neck abscesses, mastoiditis, labyrinthitis, hearing loss meningitis, brain abscesses, and sigmoid sinus thrombosis [2, 3, 12–16]. Proper and timely management in acute phase should reduce the incidence of developing complications [17].

However, there are still a large number of patients delaying in getting timely and proper management of their disease mainly due to low awareness coupled with financial constraints. Many studies show that patients with complicated CSOM belong to a lower socioeconomic status and had low awareness of complications of CSOM [15–17]. This is also true in Rwanda where low awareness and financial constraints were reported to be a barrier to care seeking for ear infections in children in a community survey [18].

We conducted a hospital based study to establish the factors resulting in delays for care seeking for CSOM as well as the characteristics of patients with delayed presentation at a referral hospital in Rwanda.

## 2. Materials and Methods

### 2.1. Study Design

The study was a cross-sectional descriptive study. It enrolled 109 patients who consulted ENT Department at the national referral hospital from 1 August to 22 December 2015.

### 2.2. Inclusion Criteria and Exclusion Criteria

The study population included all patients visiting the ENT Department of the referral hospital and diagnosed with CSOM for the first time. Delayed care seeking was defined as seeking treatment 6 months after onset of symptoms. We excluded patients who had been treated for CSOM at this hospital before and also patients diagnosed with other types of otitis media.

### 2.3. Sample Size

Our sample size was 109 patients obtained using the formula below:

(1) $$\begin{array}{c} n=\frac{z2\times p\left(1-p\right)}{d2} \end{array}$$

We estimated a prevalence of CSOM of 7.65% in the ENT Department using clinical records. The desired confidence interval was 95% and allowed a margin of error of 5%.

### 2.4. Data Collection

All the patients fulfilling the inclusion criteria were included in the study. Data was collected by the principle investigator. Predesigned questionnaires were used to collect data on the process of seeking treatment for disease. Information collected included demographics, being affiliated to a health insurance, presenting symptoms, duration between onset of symptoms and care seeking, where they sought treatment, and reasons for delayed presentation.

### 2.5. Data Analysis

The data entry and statistical analyses were performed using the SPSS 16.0. Comparison of categorical variables was performed using the chi-square test. The limit of significance was set at p < 0.05.

### 2.6. Ethical Consideration

Research proposal was presented in the ENT Department at the national referral hospital for approval and thereafter permission was sought from Research and Ethics Committee of Kigali University Teaching Hospital (KUTH) where the study was going to be conducted. The patients gave written informed consent to participate in this study. If a patient could not give consent, their guardian/parent gave consent on their behalf. Confidentiality was ensured for all collected data, during both data collection and analysis.

## 3. Results

A total of 109 patients were diagnosed with CSOM during the study period. Of these 97 (88.9%) patients fulfilled the criteria for delayed presentation and were included in this study. The age range was 3 – 72 years (SD =15.1, mean 27).

Our study population comprised 58 females and 39 males, female to male ratio of 1.5:1. The predominant age range was 21-30 years (39.2%). More than half, 58.8% (n=57), of study population resides in the rural area while students accounted for 46.4% (45) of our study subjects. Concerning education level, more than a half, 54.6%, was in secondary school; other completed secondary schools. Almost all the study population, 99%, had health insurance.  Table 1 gives further detail.

While all enrolled patients had ear discharge, those presenting within 6 weeks of onset reported no pain nor hearing loss. However, patients with these complaints gradually increased as the duration of the complaints increased. This difference was statistically significant.  Table 2 elaborates these findings.

Considering the complaint among those with delayed care seeking, the majority, 48.5% (n=47), complained of hearing loss, 42.2% (n=41) had active ear discharge while 9.3% (n=9) had ear pain as shown in Table 3.

Comparing use of traditional medicine and delayed care seeking, results show that more than a half of study population and those with delayed presentation used traditional medicine before seeking treatment at our facility at 56.9% (n=58) and 33.3 (n=4), respectively, as shown in Table 4. This was statistically significant.

Lack of awareness was the most recurrent barrier that resulted in delays in care seeking as reported by 90.7% (n=88) of our participants. Other barriers included service delivery and financial constraints by 34% (n=33) and 22.7% (n=22), respectively. These barriers included delays in getting a referral from a lower level health facility and distance barriers thus causing health seeking to be costly or simply inaccessible.  Table 5 shows these findings further.

## 4. Discussion

To the best of our knowledge, this is the first study conducted in Rwanda describing reasons for late care seeking for CSOM. This is a common condition and comparative studies show that majority of people with long standing CSOM or those with ear infections globally reside in developing countries [2, 16] and specifically in rural areas [19, 20].

During the study period, we enrolled more females than males with delayed presentation for care seeking. While a previous study in Rwanda showed no significant difference between CSOM among both sexes in children, other studies report that CSOM affects more females than males [15, 21, 22]. However, this may be true for Rwanda given the consequences of genocide of 1994 and the current population where females outnumber males [23]. In contrast, other studies show a male preponderance [1, 24].

The most frequently affected age group is the productive age and worse still more of those affected are students. Perhaps, this justifies their inability to seek treatment early due to barriers faced. Delaying treatment is a significant cause of morbidity and mortality. Up to 90% of hearing impairment in developing countries is caused by CSOM [2]. This underscores the burden of CSOM and its implication on hearing loss, productivity, global burden of disease, and cost on the health system and the economy [25].

The main cause for delayed care seeking among our patients was lack of awareness on nature, progression, and treatment of CSOM. In the current study, 90.7% of patients with delayed presentation reported that they had little or no knowledge about CSOM. Various studies have shown that lack of awareness leads the patients to delay care seeking. This is true not only in Rwanda [18] but also in other regions [26, 27]. Chandrashekharayya SH. et al. (2014) studying the level of awareness about CSOM showed that the patients with CSOM developed complications because of lack of awareness of the disease [15]. Moreover, the painless nature of CSOM causes patients to defer care seeking for later [28].

Our study showed that financial constraints contribute to delays in care seeking. Long distances to a health facility require that a patient pays a fee to be transported regardless of whether they have a medical cover. This has been reported as barrier in a community based study [18]. However, barriers encountered at health facilities were also reported. Shortage of qualified staff, long periods of waiting for services, and well as unsatisfactory services were reported in our study and in other studies [28–30].

Up to 59.8% of patients who delayed in seeking treatment at a health facility had used traditional medicine. Low awareness and beliefs, coupled with financial constraints and prohibitive health system factors, are associated with tendency to seek traditional treatment as shown in our study and other studies [27, 31]. This practice in turn results in delays in care seeking at a medical facility given the beliefs of the patients that CSOM is only treated by alternative therapies [18, 31] as shown by the results of our study.

## 5. Conclusion

Majority of patients with delayed care seeking were young adults. More than half were from rural areas. Knowledge regarding CSOM is still low and majority of participants preferred to use traditional medicine before consulting the health systems. Consequently, low awareness of CSOM and seeking traditional treatment are the main causes of delayed care seeking. There is a need to increase awareness among the population on CSOM in a bid to curb avoidable complications such as hearing impairment which were common in this study. Moreover, healthcare workers in primary healthcare facilities should be trained for management of ear infections and encouraged to refer patients to higher levels when the ear infection does not respond to treatment.

## Figures and Tables

**Table 1:** Characteristic of patients with delayed care seeking for treatment of CSOM.

		**Late n=97**	**Percent**
**Age of patient**	<10 years	11	11.3
	11-20 years	25	25.8
	21-30 years	38	39.2
	>31 years	23	23.7
**Gender**	Male	39	40.2
	Female	58	59.8
**Residence**	Rural area	57	58.8
	Urban area	40	41.2
**Occupation **	Paid employee/Office worker	25	25.8
	Self-employed/Commerce	27	27.8
	Student	45	46.4
**Education level **	Complete primary/none	44	45.4
	Post primary	53	54.6
**Insurance **	Health insurance	96	99
	None	1	1.0

**Table 2:** Patients' complaints compared to duration from onset to care seeking.

	**Duration of complaints**				
**Complaint**	**< 6 weeks (n=2)**	**6 weeks-6 months (n=10)**	**6-12 months (n=12)**	**> 12 months (n=85)**	**Total (n=109)**
**Ear Discharge**	2 (1.8%)	7 (6.4%)	7 (6.4%)	34 (31.2%)	50 (45.9%)
**Hearing loss**	0	2 (1.8%)	4 (3.7%)	43 (39.4%)	49 (44.9%)
**Pain **	0	1 (0.9%)	1 (0.9%)	8 (7.3%)	10 (9.2%)

Pearson chi=0.003. Pain^*∗*^  mean headache or otalgia.

**Table 3:** Patients' complaint and duration.

	**Duration of complaint**		
**Complaint**	**6-12 months**	**>12 months**	**Total (n=97)**
**Ear discharge**	7 (7.2%)	34 (35.1%)	41 (42.2%)
**Hearing loss**	4 (4.1%)	43 (44.3%)	47 (48.5%)
**Pain**	1 (1%)	8 (8.2%)	9 (9.3%)

**Table 4:** Time and duration for patients that used traditional medicine.

	**Late n=97**		**Early n=12**	
	**Frequency**	**%**	**Frequency**	**%**
Used traditional Medicine	58	59.8	4	33.3
Not applicable	39	40.2	8	66.7
Total	97	100	12	100

**Table 5:** Barriers to early care seeking.

		**Presentation time**			
		**late n=97**	**early n=12**	**Total**	**p-value**
Service	Yes	33 (34%)	3 (25%)	36 (33%)	0.747
	No	64 (66%)	9 (75%)	73 (67%)	
Financial	Yes	22 (22.7%)	3 (25%)	25 (22.9%)	0.988
	No	75 (77.3%)	9 (75%)	84 (77.1%)	
Knowledge	Yes	88 (90.7%)	5 (41.7%)	93 (85.3%)	0.001
	No	9 (9.3%)	7 (58.3%)	16(14.7%)	

## Data Availability

Data is available upon request.
